# Characteristics of practitioners in a private managed behavioral health plan

**DOI:** 10.1186/1472-6963-12-283

**Published:** 2012-08-28

**Authors:** Sharon Reif, Maria E Torres, Constance M Horgan, Elizabeth L Merrick

**Affiliations:** 1 Institute for Behavioral Health, Heller School for Social Policy and Management Brandeis University, Waltham, MA, USA

**Keywords:** Health plans, Managed care, Treatment providers, Demographics, Substance abuse, Mental health

## Abstract

**Background:**

Little is known about the practitioners in managed behavioral healthcare organization (MBHO) networks who are treating mental and substance use disorders among privately insured patients in the United States. It is likely that the role of the private sector in treating behavioral health will increase due to the recent implementation of federal parity legislation and the inclusion of behavioral health as a required service in the insurance exchange plans created under healthcare reform. Further, the healthcare reform legislation has highlighted the need to ensure a qualified workforce in order to improve access to quality healthcare, and provides an additional focus on the behavioral health workforce. To expand understanding of treatment of mental and substance use disorders among privately insured patients, this study examines practitioner types, experience, specialized expertise, and demographics of in-network practitioners providing outpatient care in one large national MBHO.

**Methods:**

Descriptive analyses used 2004 practitioner credentialing and other administrative data for one MBHO. The sample included 28,897 practitioners who submitted at least one outpatient claim in 2004. Chi-square and t-tests were used to compare findings across types of practitioners.

**Results:**

About half of practitioners were female, 12% were bilingual, and mean age was 53, with significant variation by practitioner type. On average, practitioners report 15.3 years of experience (SD = 9.4), also with significant variation by practitioner type. Many practitioners reported specialized expertise, with about 40% reporting expertise for treating children and about 60% for treating adolescents.

**Conclusions:**

Overall, these results based on self-report indicate that the practitioner network in this large MBHO is experienced and has specialized training, but echo concerns about the aging of this workforce. These data should provide us with a baseline of practitioner characteristics as we enter an era that anticipates great change in the behavioral health workforce.

## Background

The private sector is responsible for a substantial portion of treatment for mental and substance use disorders, with private insurance accounting for about 24% of mental and substance use disorder expenditures in 2005 [1]. While that proportion had been projected to decrease somewhat by 2014 [2], it is now likely that the role of the private sector will increase due to the recent implementation of federal parity legislation and the inclusion of behavioral health as a required service in the insurance exchange plans created under healthcare reform.

Most private health plans carve out treatment for mental and substance use disorders to specialty managed behavioral healthcare organizations (MBHOs) [3]. Health plans and MBHOs use many techniques to manage care, such as utilization review and prior authorization. One approach that has received little study is the MBHO’s use of networks of approved or credentialed treatment practitioners who provide office-based treatment (“in-network practitioners”) in order to address quality and cost goals. Networks also allow plans to ensure the availability of a variety of practitioners in terms of demographics, location, and skills [4-6].

Most literature about managed care and practitioners focuses on their attitudes and concerns about the impact of managed care on professional decision-making, income, treatment options, types of patients and the changing role of psychiatrists [5,7-14]. However, little is known, beyond practitioner types, about the practitioners in MBHO networks who are treating mental and substance use disorders for privately insured patients. The few published workforce studies provide snapshots of practitioner networks [15-17] or facility-based staff [18-20], but do not delve further into demographics, years of experience or specialized expertise. A recent analysis, conducted with the same data used here, found that other than nurses, the network of behavioral health practitioners was distributed fairly evenly across practitioner types, with slightly more psychologists and social workers than psychiatrists and counselors [21]. Further, individual therapy is the predominant type of outpatient treatment, even by psychiatrists [21]. About half of these practitioners reported expertise in treating major psychiatric disorders, and about one third reported expertise in treating substance use disorders [21].

The 2010 healthcare reform legislation has highlighted the need to ensure a qualified workforce in order to improve access to quality healthcare, and provides an additional focus on the behavioral health workforce. Concerns have also been raised about the aging of the workforce [22,23] and about practitioners “opting-out” of insurance networks altogether. With need for a more nuanced understanding of behavioral health treatment in the private sector, it is important to obtain a more current view of these practitioners than is presently available. The gaps in the literature are large, and this study aims to address several: we consider practitioner patterns nearly a decade later than the most recent studies, as managed care is more prevalent but prior to the advent of health care reform which is expected to bring additional change to the behavioral health workforce; describe the demographics of these practitioners; expand the focus beyond the role of psychiatrists; and consider experience and training. More precisely, to expand our understanding of private sector treatment, this study uses administrative data to examine the practitioner types, years of experience, specialized expertise, and demographics of in-network practitioners providing outpatient care in one national MBHO.

## Methods

Data were from a large national managed behavioral healthcare organization (MBHO) for calendar year 2004. This analysis is a follow-up to the study noted above [21]. Two sources of data were used: (1) practitioner credentialing data for in-network practitioners, based on information provided by practitioners when they applied for inclusion in the MBHO’s network; and (2) claims files for patients, to identify whether an in-network practitioner saw any patients in this MBHO in 2004. Secondary data analysis was limited to all practitioners in the network who submitted at least one outpatient claim in 2004 (N = 28,897). The study received Institutional Review Board approval from Brandeis University.

Practitioner characteristics include age, gender, and bilingual status. Practitioner race/ethnicity was not required for credentialing with this MBHO, thus was omitted by 72% of practitioners, and is not reported here. Education and licensure were identified. Practitioners were coded into mutually exclusive practitioner types based on their licensure: psychiatrist, psychologist, licensed social worker, nurse (advanced registered nurse practitioner, registered nurse, or registered nurse clinical specialist), or master’s level/licensed counselor. A small proportion of practitioners who fell into multiple practitioner types were coded to the highest category, in the order noted above.

Years of post-training experience was directly reported by 85% of practitioners, and was otherwise imputed using degree type and year of graduation. Specialized training was ascertained by the item: “Please check the areas [major psychiatric, alcohol/drug, children 0-12, adolescents 13-17, and marital/family] in which you have at least 1500 hours of training and experience and wish to provide services. Your experience should be evidenced in your work history.” This analysis focuses on expertise in treating children and adolescents, as well as marital and family counseling.

Univariate and bivariate analyses describe the practitioners. Statistical significance was determined with chi-square tests and t-tests, with pairwise comparisons across practitioner types. Corrections were made for multiple pairwise comparisons by using the Bonferroni correction. All statistical analyses were conducted with SAS v9.1.

## Results

The MBHO had 28,897 practitioners in-network who saw patients with mental or substance use disorders in 2004 (Table1). Female practitioners comprise over half of the practitioner network (55.3%). However, the gender distribution varies greatly by practitioner type, with psychiatrists having the fewest women (23.0%) and social workers, nurses, and counselors having the most women (72% or more). Nearly all nurses were women (85.3%).

**Table 1:** Practitioner characteristics by practitioner type

	**Percent of Practitioners**					
	**Total**	**Psychiatrist**	**Psychologist**	**Social Worker**	**Nurse**	**Counselor**
**N**	28,897	5,703	9,191	8,248	559	5,184
**Percent**	100.0	19.6	31.8	28.7	1.9	18.0
Gender*						
Female	55.3	23.0	49.2	71.9	85.3	72.3
Male	38.0	63.1	46.0	23.3	7.0	23.1
Unknown	6.6	13.9	4.8	4.7	7.7	4.6
Bilingual*						
Yes	12.1	23.3	10.1	8.6	4.1	9.5
No	86.4	74.3	88.4	90.0	94.8	89.7
Unknown	1.5	2.5	1.5	1.4	1.1	0.8
Age*						
<45	19.0	23.1	16.2	18.2	12.9	21.2
45-49	13.6	15.4	12.4	13.3	13.2	14.0
50-54	20.3	16.3	21.6	20.9	25.8	20.8
55-59	21.1	14.8	23.4	22.4	24.2	21.8
60-64	12.9	10.2	13.6	14.0	15.4	12.6
> = 65	9.3	11.2	9.8	8.5	5.7	7.9
Unknown	3.9	9.0	3.1	2.6	2.9	1.7
Mean Age (SD)**	52.7	51.9	53.5	52.7	53.1	51.9
	(9.8)	(10.6)	(9.4)	(9.6)	(8.7)	(9.8)

Sections within columns add to 100%.

*p < .001 for all pairwise comparisons of practitioner types.

**Mean age p < .001 except not significant for psychiatrist vs. counselor, and nurse vs. psychologist and social worker.

Twelve percent of practitioners report being bilingual or multilingual, representing 37 different languages (Table1). The most frequently cited other languages were Spanish, French, German and Hindi. Psychiatrists were significantly more likely to be bilingual (23.3%) than were other practitioners, and nurses were least likely (4.1%).

Two-thirds of practitioners were over 50 years old (Table1), with a mean age of 52.7 (SD = 9.8). Although the mean age is similar across the practitioner types, there was variation in age distributions. More psychiatrists (38.5%) and counselors (35.2%) were younger than 50 compared to other practitioner types (26.2% to 31.6%). Yet, psychiatrists have the most post-training experience by far (Table2), with 41.7% reporting more than 20 years of experience, and counselors the least, with 54.2% reporting fewer than 10 years of experience. On average, network practitioners report 15.3 years of experience (SD = 9.4).

**Table 2:** Practitioner years of experience and specialized expertise by practitioner type

	**Percent of Practitioners**					
	**Total**	**Psychiatrist**	**Psychologist**	**Social Worker**	**Nurse**	**Counselor**
Post Training Experience (years)*						
0 to 5	14.6	6.1	11.9	13.7	17.4	29.9
6 to 10	19.9	12.9	16.9	22.3	18.8	24.3
11 to 15	20.1	15.6	19.9	22.8	12.0	21.8
16 to 20	15.1	12.8	18.0	16.1	10.0	11.6
21 to 25	12.4	13.1	15.5	12.2	9.5	6.9
25 or more	14.2	28.6	14.4	10.0	28.4	3.3
Unknown	4.6	10.9	3.5	3.0	3.9	2.3
**Mean Post-Training**	15.3	20.4	16.0	14.3	17.6	10.5
Experience (SD)*	(9.4)	(11.1)	(8.8)	(8.2)	(11.9)	(7.0)
**Specialized Training**						
Children 0-12*	41.0	25.0	48.6	42.0	23.6	45.5
Adolescents*	61.1	40.9	69.0	64.2	43.1	66.6
Marital/Family*	66.8	23.9	77.1	77.3	46.5	81.5

Sections within columns add to 100% only for post training experience categories.

*p < .001 for all pairwise comparisons of practitioner types.

A high proportion of practitioners reported specialized expertise (Table 2). Marital/family expertise was reported by the most, about two-thirds of practitioners. Specialized expertise in treating children was reported by 41.0% of practitioners and 61.4% reported specialized expertise in treating adolescents. When stratified by practitioner type, the proportions of practitioners reporting specialized expertise also vary. Psychiatrists and nurses were least likely to report specialized expertise in children, adolescents, or marital/family. Counselors report expertise in children, adolescents and marital/family at comparable levels to psychologists and social workers.

## Discussion

These data provide important insights into the current workforce, as well as a window into possible future challenges. They support the findings of national reports in terms of the age and gender distribution of the practitioner workforce [22,23], with the majority of these practitioners being women, and provide evidence that the workforce is aging, with median age here of 53 years old. In addition to reflecting national findings, the gender distribution and years of experience for each type of practitioner in the network, while varying by type, reflect the distributions reported by organizations representing those professions [24,25].

When the data are considered by practitioner type, a few distinctions are evident compared to national data in similar time periods [26]. This practitioner network has a lower proportion of male counselors than is seen in national data (23% vs. 35% nationally), and a higher proportion of male social workers (23% vs. 18%), while psychiatrists, psychologists, and nurses have comparable gender distributions across these data and national data [26]. This network has lower proportions of practitioners in the youngest age range (<45), except for psychiatrists and nurses, and lower proportions of the oldest age range (65+) for all practitioner types. National data indicate that 17% of psychiatrists, 27% of psychologists, 36% of social workers, 12% of nurses and 40% of counselors are under the age of 45, and that 32% of psychiatrists, 17% of psychologists, 7% of social workers, 6% of nurses, and 11% of counselors are older than 65 [26].

The gender distributions also indicate the continuing predominance of women in the traditionally female-staffed fields of nursing and social work. Women also are in the professions with fewer years of experience overall, perhaps due to absence from the workforce related to child-rearing, or to later entry into these women-dominated professions for the same reason or as part of mid-career shifts. Our data, however, cannot test these theories. Lower average experience in certain professions may also be due to higher burnout. The literature suggests that turnover is high among substance abuse and mental health counselors [22,23] and among nurses [27] due to demanding working conditions and, particularly for counselors, low pay. Psychologists and psychiatrists have much more time invested in their educational requirements, and receive higher pay for their work, thus it seems reasonable that they may be more likely to remain in those careers.

The aging of the workforce has been highlighted as a concern by SAMHSA, the Annapolis Coalition on the Behavioral Workforce [23], the Institute of Medicine [22] and others. These data demonstrate that across practitioner types the workforce is aging, and suggest that at the same time there is not an influx of younger providers coming in to the field to replace the older providers as they retire, with only 19% of practitioners under the age of 45. Given that these are professions where a great deal of training and skills develop and are sharpened “on the job”, the skills, expertise, and institutional knowledge of the older and more experienced workers may not be passed on. Without an influx of younger/new providers, the aging of the workforce creates a vacuum in respect to both the future leadership of the field and the human capital required to provide the highest quality of care.

While age may affect the sufficiency of the workforce overall, bilingual capability is a practitioner characteristic that may directly affect access to care. About 20% of the U.S. population speaks a language other than English, and nearly one fourth of those report their English-speaking ability as “not well” or “not at all” [28]. The proportion of bilingual practitioners reported here, at 12%, is somewhat less than the U.S. population, although nearly one fourth of these psychiatrists are bilingual. The higher proportion of bilingual psychiatrists may reflect the estimated 25% of office-based physicians who are international medical graduates [29]. Although the primary languages of these practitioners reflect some of the most common languages in the U.S., these data cannot indicate how well patients and practitioners are matched based on their preferred language or if the provider’s bilingual status translated into improved access to care.

Sufficient ability to treat children and adolescents in need of behavioral health treatment has been highlighted as a concern for some time [22,30-34]. Although not all practitioners in this MBHO demonstrate this expertise as part of their credentialing application, it is fairly common, with two out of five practitioners expert in treating children and three out of five expert in treating adolescents. These proportions are lower for psychiatrists, where concerns are often noted about sufficient capacity [31,32], but one fourth of these psychiatrists report expertise in treating children, and even more for adolescents. However, we cannot determine from these data if and how that translates to sufficient access; for instance, these practitioners with specialized expertise in treating children or adolescents might all be based in urban areas, with rural shortages particularly noted in other research [31,32], thus potentially leaving gaps for some patients.

A few limitations should be noted. Training and expertise are self-reported by the practitioners, and generally not verified by the MBHO. Although this study contributes to our understanding of practitioners and treatment in private MBHOs, it could not examine the specific content or quality of treatment offered, and little is known about the patients themselves, such as comorbidities or severity. Broader issues of access to care, such as geographic access, authorization for treatment, or whether practitioners are accepting new patients are also key to consider in future studies. Patients in private health plans may opt to see out-of-network practitioners, paid for by the plan or out of pocket, who are not considered here. This study cannot address concerns about whether practitioners are opting out of insurance networks, and which practitioners are doing so. These data stem from only one MBHO, but it is a national one and results reflect other findings in the literature; further, there is no reason to expect that this network would be different than others. In addition, most providers are in multiple networks [35], so these characteristics should be mirrored elsewhere. Although collected in 2004, these data provide a baseline description of the behavioral health workforce prior to the changes expected as parity and health care reform are implemented. Despite these potential limitations, we find that on the whole, practitioners in this MBHO have substantial experience, and a large proportion report specialized training.

## Conclusions

This study describes the characteristics of practitioners who provide the outpatient treatment that privately insured patients with mental and substance use disorders receive. This MBHO network appears to represent a range of practitioner types and demographics, with use of appropriately qualified practitioners with substantial experience on average. These findings do not lend credence to the concern that MBHOs are using inexperienced or poorly qualified practitioners, and indicate that there is a solid, experienced core of practitioners who accept insurance referrals.

With the advent of healthcare legislation, workforce issues will be exacerbated, with implications for the delivery of quality care by qualified providers. Prior to the passing of new healthcare legislation, all practitioner types considered here were projected to experience “average” to “much faster than average” job growth from 2008-2018 [36]. With both healthcare reform and parity legislation being implemented and increasing the focus on provision of mental health services, practitioner shortages and competition for skilled practitioners will increase, adding to the impact of retiring workers leaving the field. Strategies to counter this effect include the courting of “second career professionals”; increased efforts to retain older professionals in the workforce [37,38] which could offset the vacuum of human capital, institutional knowledge and leadership that is projected to occur; and encouraging high school and college students to consider a career in behavioral health [23]. It will also be worthwhile to consider how the increasing move to integration of primary care and behavioral health services will affect the behavioral health workforce.

While there is room for improvement, in terms of specialized training to best address the needs of patients with substance use and mental disorders, this practitioner network seems to be highly experienced and is well represented by practitioners with specialized training, with positive implications for patient care. Concerns about insufficient capacity to treat children and adolescents may be alleviated in part by using non-psychiatrist practitioners who are available in greater numbers and still report expertise for these populations. Further research is needed to see if and how this reported expertise translated to access to care, and quality of care. These data should provide us with a baseline of practitioner characteristics as we enter an era that anticipates great change in the behavioral health workforce.

## Competing interests

The authors declare that they have no competing interests, financial or non-financial.

## Authors’ contributions

SR conceived of the study and led the study design, analysis and writing. MET, CMH, and ELM contributed to design and analytic decisions, and participated in the writing and revisions of the manuscript. All authors reviewed and approved the final manuscript.

## Pre-publication history

The pre-publication history for this paper can be accessed here:

http://www.biomedcentral.com/1472-6963/12/283/prepub
